# Percutaneous thrombectomy of fat embolism in-transit

**DOI:** 10.1016/j.jvscit.2024.101702

**Published:** 2024-12-02

**Authors:** Erin Cihat Saricilar, Cartan Costello, Laurencia Villalba, Alexander Misono

**Affiliations:** a Wollongong Hospital, Wollongong, New South Wales, Australia; b University of Wollongong, Wollongong, New South Wales, Australia; c Vascular Care Centre, Wollongong, New South Wales, Australia; d Hoag Hospital Irvine, Irvine, CA

**Keywords:** Fat embolism, Large-bore thrombectomy, Pulmonary embolism

## Abstract

We report the case of a previously independent 82-year-old female who experienced acute hemodynamic and respiratory deterioration requiring inotropic support due to a fat embolism during revision hip arthroplasty. Computed tomography pulmonary angiography demonstrated fat embolism, and transesophageal echocardiogram showed evidence of right ventricle strain and fat embolism in-transit in the right heart, as well as a moderate patent foramen ovale. Under transesophageal echocardiogram and intravascular ultrasound guidance, the Inari FlowTriever thrombectomy device was used successfully to retrieve the fat embolism with immediate hemodynamic improvement, no complications, and uneventful recovery.

Fat embolism (FE), occurring in 90% to 95% of patients with long bone fractures following trauma or orthopedic procedures involving long bones, is common, and its clinical presentation may be either subtle or life-threatening.^1^ FE syndrome (FES) is a poorly defined clinical phenomenon estimated to occur in 1% to 23% of long bone fractures,^2^ which can result in pulmonary failure, neurologic symptoms, multisystem dysfunction, and even death.^1^^,^^3^

Estimated mortality rates from FES vary between 7% and 36%, with much higher rates reported from autopsy studies.^2^

Similarly, right heart thrombi in the context of pulmonary embolism (PE) is a marker of severity,^4^ with a mortality rate up to 21% in 14 days,^5^ and treatment escalation is advocated for select patients. Open surgical thrombectomy or catheter-directed therapy have a role in patients with rapid hemodynamic deterioration, clot in-transit (CIT) (defined as embolic material within the right heart that is not attached to any intracardiac structure) when an underlying right-to-left shunt is present.^6^

We present a case of fat PE with concurrent right heart emboli in an unstable elderly trauma patient presenting with a concurrent subdural hematoma and a moderate patent foramen ovale (PFO) who underwent percutaneous large-bore aspiration thrombectomy. The patient consented to this report being written and published.

## Case report

An 82-year-old patient living independently at home was brought into the emergency department of a large tertiary referral hospital by ambulance following a mechanical fall. On her background, she had bilateral hip replacements, rheumatoid arthritis on methotrexate, and history of a left popliteal artery stent on life-long low-dose aspirin. Computed tomography (CT) demonstrated a periprosthetic fracture of the right hip (Vancouver B), and CT of the brain showed a subdural hematoma, which was managed conservatively by withholding antiplatelets and anticoagulants.

Five days later, the patient underwent a revision right hip arthroplasty.

During surgery, the patient experienced severe hypotension and hypoxia, most noticeable but not confined to the period surrounding reaming of the femoral canal. She was managed with intravenous fluid resuscitation to replace high-volume blood loss with two units of packed red blood cells and two liters of plasmalyte crystalloid fluid, followed by an additional liter of 4% albumin fluid bolus and vasopressor therapy requiring a metaraminol bolus intraoperatively, followed by a noradrenaline infusion running at 0.2 mg/hr. An intraoperative transthoracic echocardiogram (TTE) did not reveal any right heart dilatation or strain, potentially masked by the hypovolaemic shock. The operation was successfully completed. The patient was taken to the intensive care unit (ICU) ventilated and on multiple inotropes in a state of shock. Due to the high clinical suspicion of FE, a CT pulmonary angiography (CTPA) was performed. It showed multiple fat-attenuation filling defects in segmental and subsegmental arteries with a Miller index of 8 and a mildly dilated pulmonary trunk. A TTE in ICU that was performed following adequate fluid resuscitation by a specialized echocardiographer focusing on right heart function was noted to be technically difficult due to severe pectus excavatum; however, it showed a normal size right ventricle (RV), progressive right heart strain with reduced right ventricular contractility and elevated right ventricular systolic pressure of 45 mmHg, which was becoming more apparent with fluid resuscitation, and an echogenic mass adherent to the tip of the central venous catheter (CVC) in the right internal jugular vein (IJV). Supportive measures continued, but due to the concurrent subdural hematoma, anticoagulation was not started, and expectant management followed. This was based on discussion with neurosurgery and hematology, where there is equipoise in the literature about the risk of progression of intracranial hemorrhage when anticoagulation is administered,^7^ with some large studies demonstrating no venous thromboembolism (VTE) benefit with increased hemorrhage expansion risk,^8^ whereas others demonstrate the opposite.^9^ Considering all the concurrent medical and surgical challenges with the underlying comorbidities, the multiple disciplines involved elected to continue conservative management with close observation.

**Fig 1:**
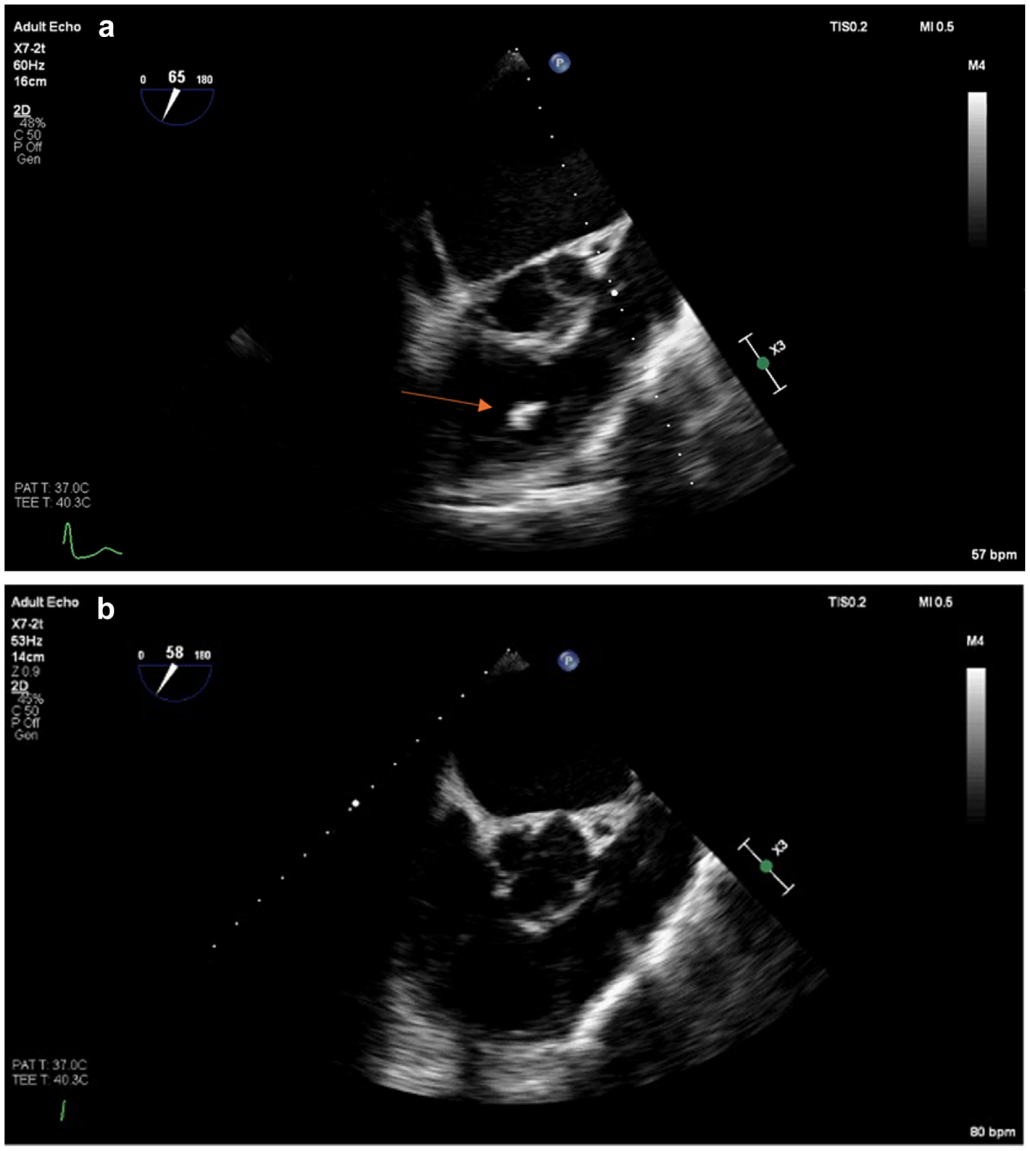
**A,** Clot-in-transit (CIT) within the right atrium as demonstrated on transesophageal echocardiogram (TEE) and marked with an *orange arrow*. **B,** TEE performed 1 day after the suction thrombectomy demonstrating that the CIT is no longer present in the right atrium or right ventricle (RV).

**Fig 2:**
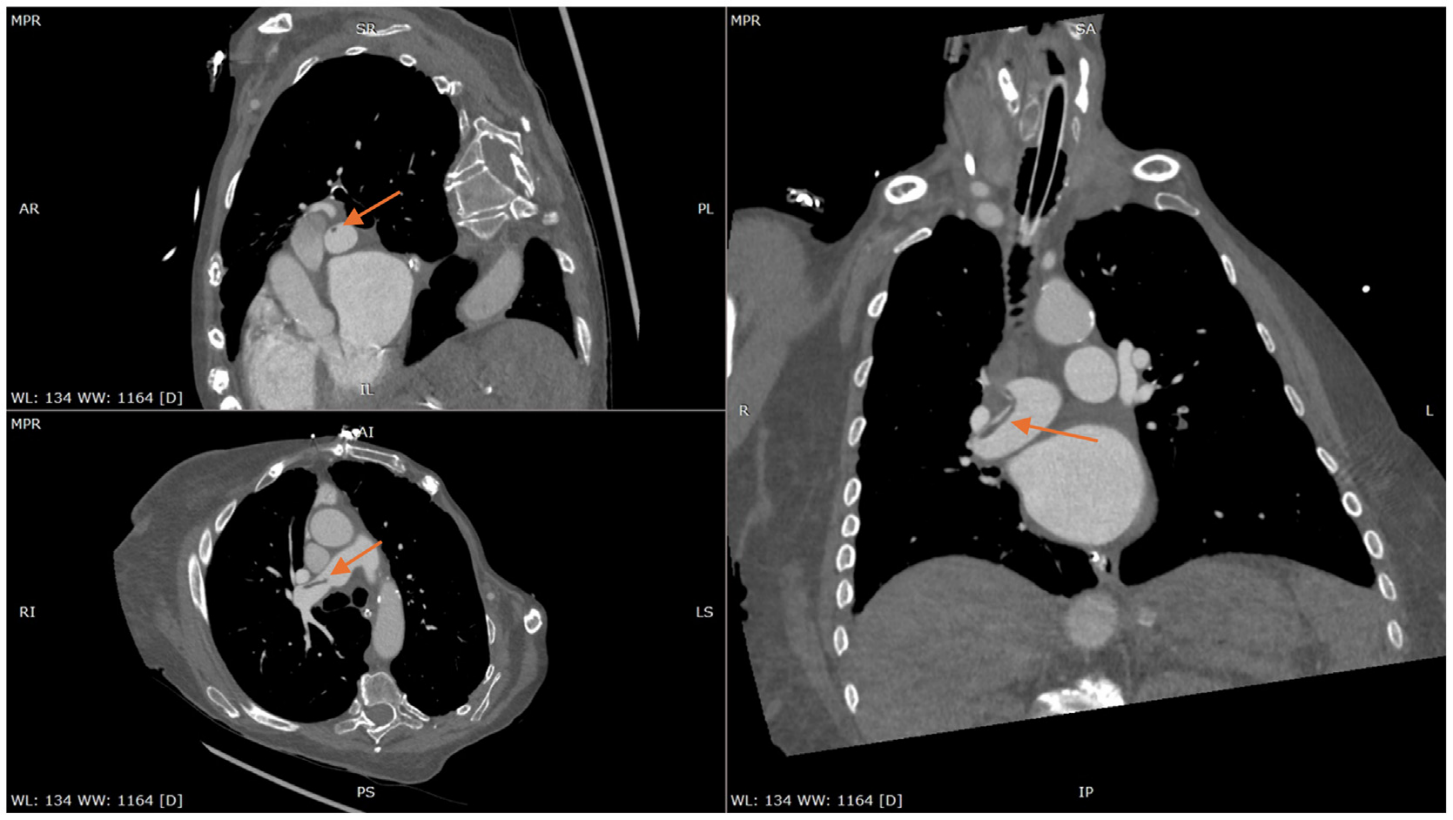
Computed tomography pulmonary angiogram (CTPA) demonstrating small pulmonary emboli (PE) presumed to be fat embolism (FE) based on the Hounsfield units (HUs), which has been rotated with three-dimensional multiplanar reconstruction to better visualize the FE, which is marked with *orange arrows*.

The next morning, a transesophageal echocardiogram (TEE) demonstrated worsening right heart strain, moderate tricuspid regurgitation at 3 m/s, a CIT through the RV and right atrium as demonstrated in Fig 1, *A* (with Fig 1, *B* demonstrating resolution of the CIT postoperatively), and a moderate PFO with a significant right-to-left shunt. Fig 2 demonstrates a PE that was presumed to be an FE based on the Hounsfield units. Fat can be distinguished from VTE because the attenuation value of fat is between −50 Hounsfield units (HU) to −150 HU, whereas VTE is 33 HU.^10^

### Decision-making process

The patient was not improving despite a further two units of blood product (totaling four units) and a further liter of crystalloid resuscitation (totaling three liters), which was making the RV strain more evident on the TTE.

The intrapulmonary embolic burden was low; however, the patient was deemed to have suboptimal physiological reserve, and because she was in shock and had evidence of right heart dysfunction on TOE, the pulmonary embolism response team (PERT) deemed her at high risk of deterioration because it was thought she would not tolerate further embolic insult. Additionally, the moderate PFO added risk for paradoxical embolism, which would have carried with it potentially debilitating results, as there is no lysis option. There is no risk quantification evidence of a CIT converting to a paradoxical embolism with a PFO; however, there are many case reports in the literature of this occurring. Additionally, with a moderate PFO, it was likely that the patient was likely not tolerating an even minor increase in the right heart afterload.

The multidisciplinary PERT, which included intensivists, respiratory, hematology, and vascular surgery, evaluated options, and after consulting the cardiothoracic surgeons, she was deemed not suitable for open thrombectomy due to her overall frailty. However, a percutaneous approach with large-bore thrombectomy catheters was deemed an acceptable risk.

### Procedure

The procedure was performed with assistance of intraoperative TEE and intravascular ultrasound because there was concern about being able to visualize the FE with digital subtraction angiography in the cardiac chambers and in the pulmonary tree. A bolus of intravenous heparin of 5000 IU was administered during the case, which was deemed safe after discussion with neurosurgery.

The intensive care specialist attended theater and performed the TEE, demonstrating considerable right heart strain, and confirmed a moderate PFO, and thrombotic echogenic material in the right atrium, RV, and on the tip of the central line.

Bilateral common femoral vein access was gained under ultrasound guidance, followed by 10 French Cordis AVANTI sheaths. Abbott Prostyle Perclose (Abbott Laboratories) closure devices were placed in pre-close fashion on the right side to reduce the risk of bleeding after inserting a 24 French device and to promote early mobilization postoperatively. At this point, the patient became increasingly unstable with episodes of supraventricular tachycardia and ongoing requirement of inotropic support, with noradrenaline increased to 0.32 mg/hr.

**Fig 3:**
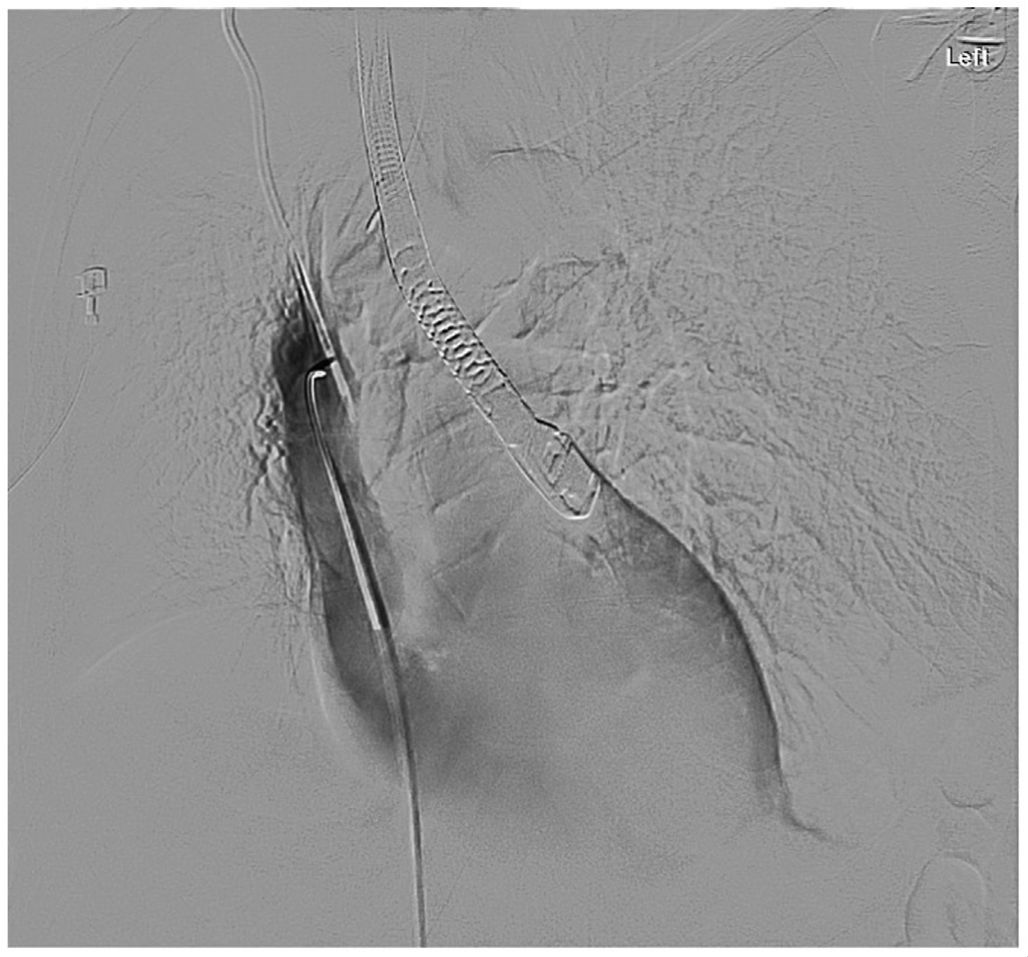
Digital subtraction imaging demonstrating the positioning of the echocardiogram probe and vascular catheters to assist in positioning to locate the clot-in-transit (CIT).

**Fig 4:**
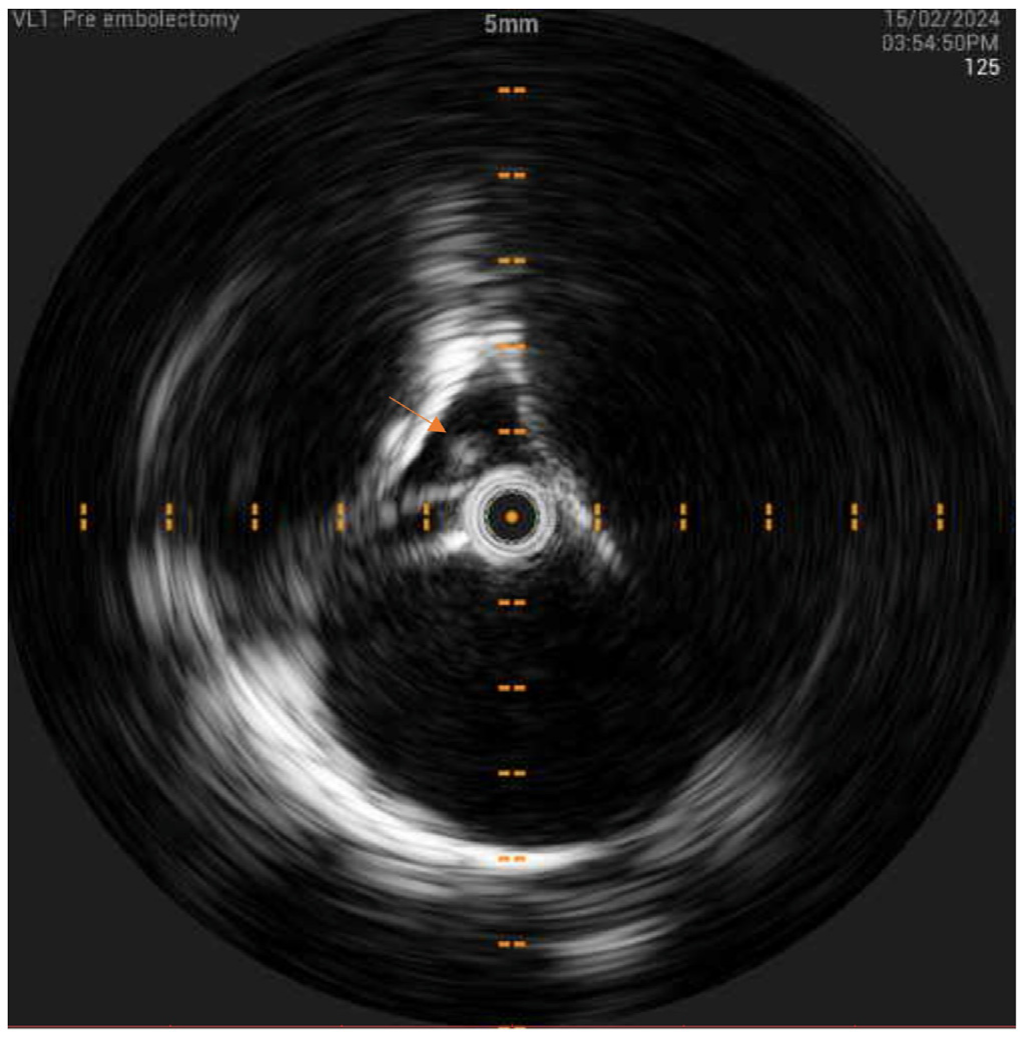
Intravascular ultrasound demonstrating thrombotic material in the right atrium demonstrated with a *superimposed arrow*.

The plan was to first remove the risk of further deterioration by removing the CIT from the right side; a wire was positioned alongside the CVC into the right IJV. Considering the patient’s hemodynamic compromise and moderate PFO, it was deemed safer to do this first rather than attempt to cross the right heart and potentially embolizing through the PFO. From the left side, another wire, followed by an intravenous ultrasound .035 catheter (Philips Volcano Visions), was also positioned in the right IJV; however, the TEE was better at demonstrating thrombus in the right atrium, RV, and over the CVC (Figs 3 and 4).

**Fig 5:**
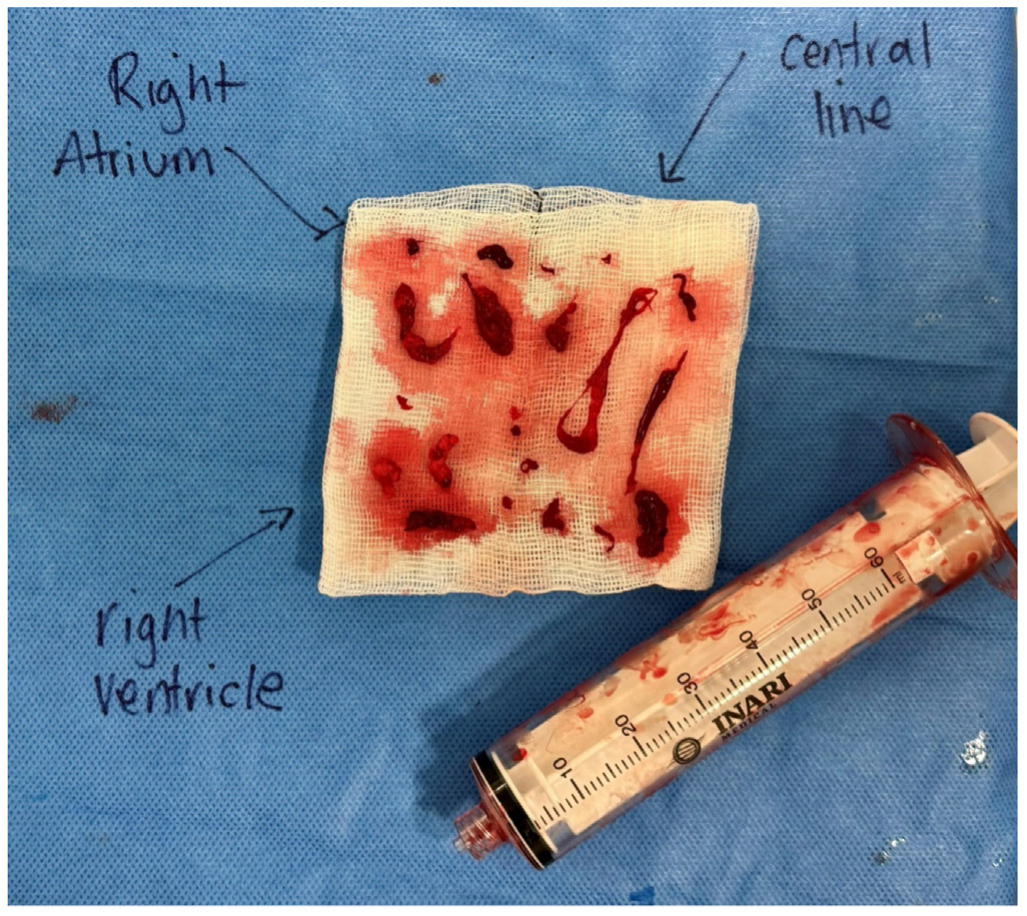
Photograph of the thrombotic material removed from the superior vena cava, right atrium, and right ventricle (RV) using the Inari FlowTriever suction thrombectomy system.

Over an Amplatz stiff wire, a 24 Fr Inari24 Introducer Sheath was inserted, followed by the FlowTriever Triever20 catheter (Inari Medical). The catheter was positioned adjacent to the thrombus with the aid of TEE. After four aspirations, each yielding fatty and thrombotic material, improvement in RV function was noted on echocardiogram. The intracardiac mass was no longer seen on TEE (Fig 1, *B*), and the thrombus adherent to the central line was also suctioned under intravascular ultrasound guidance (Fig 5), which was done to reduce the risk of progression, considering poor baseline cardiac function and a moderate PFO in the context of continuous contraindication to anticoagulation and high clinical suspicion of FEs. The Inari FlowSaver device (Inari Medical) was used after each aspiration to filter and return the aspirated blood to the patient through the introducer sheath, thereby minimizing the blood loss of the procedure. Interestingly, there was a decrease of vasopressor requirement soon after. Because the device was already in the cardiac chambers and the suction thrombectomy had been successful, an attempt to access the pulmonary arteries was considered, but because the catheter could not be navigated towards the PA due to the moderate PFO and because the patient was improving hemodynamically with reduced inotropic requirements down to 0.1 mg/hr of noradrenaline, and the intracardiac embolic material had been aspirated, reducing the risk of deterioration, a decision was made to not to proceed with suction thrombectomy in the pulmonary arteries at that stage.

On completion of intervention, the 24Fr sheath was removed from the right groin and closed with the Abbott Prostyle Perclose closure device, and the smaller 8Fr sheath from the left groin had 10 minutes of manual pressure because a hood of great saphenous vein puncture was performed, and the patient’s hemodynamics had improved significantly. Total estimated blood loss was 50 mL. Total operative time was 45 minutes. The patient was then transferred back to the ICU.

### Postoperative course

A fixed rate of 15,000 units of heparin per day was administered as three subcutaneous bolus injections, considering the balanced risk of worsening of the subdural hemorrhage and the clotting risk based on neurosurgical guidance, with strict monitoring of neurologic exam and urine output. Inotropic support was completely ceased 18 hours post procedure. The patient was extubated within 36 hours and stepped down from ICU 48 hours postoperatively, with a Glasgow coma score of 15.

Postoperative serial TTEs demonstrated no right heart strain with a right ventricular systolic pressure of 31 mmHg, normal size, and improved contractility of the RV, with resolution of tricuspid regurgitation. A repeat CTPA 48 hours after the procedure to assess the postoperative outcomes of the procedure demonstrated minor residual filling defect on the pulmonary arteries.

Postoperatively, the patient continued her low-dose aspirin and transitioned from subcutaneous heparin boluses to low-dose subcutaneous enoxaparin 20 mg for 6 weeks at day 5 postoperatively. The patient successfully underwent rehabilitation for her hip fracture and returned to her pre-morbid status at her follow-up 6 months postoperatively. The PFO was no longer evident on the postoperative TTEs because the RV strain had resolved, so further investigations and consideration for treatment were considered unnecessary.

### Histopathology

**Fig 6:**
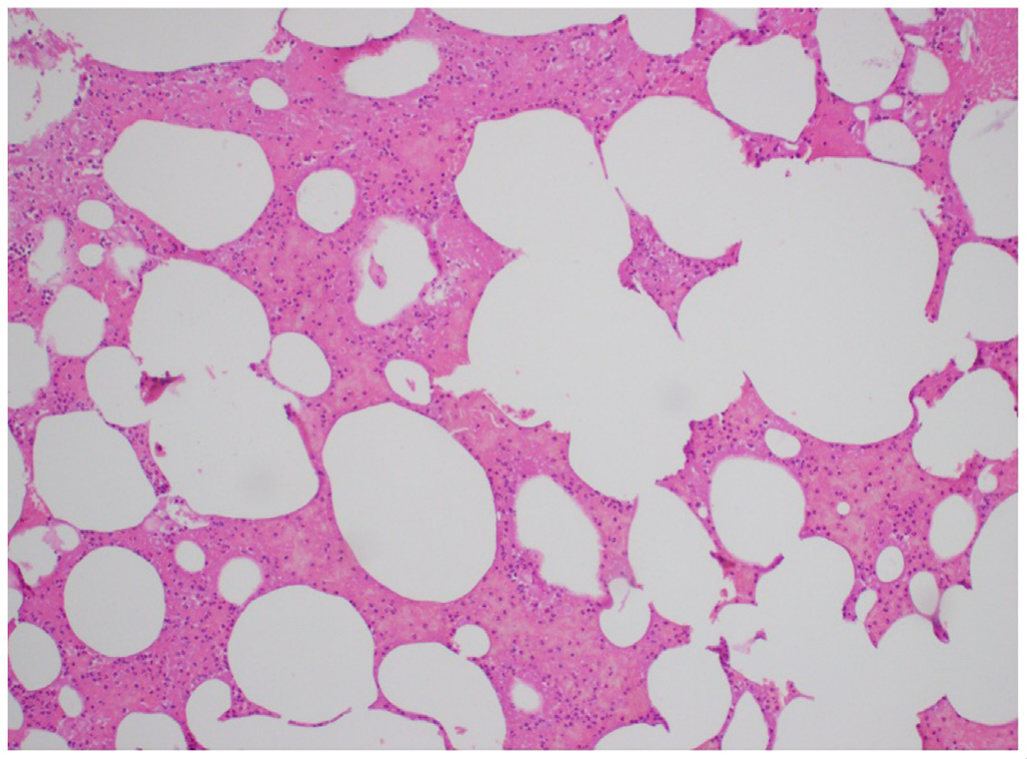
Histological slides demonstrating hemorrhage and fibrin with the empty spaces demonstrating fat vacuoles.

The material aspirated from the cardiac chambers was sent to histopathology to confirm diagnosis. The report read, “The sections show predominantly hemorrhage and fibrin admixed with these are empty spaces representing fat vacuoles, consistent with fat embolus. There are no atypical features.” Histopathological slides are demonstrated in Fig 6.

## Discussion

This case demonstrates the successful use of a large-bore thrombectomy catheter to perform percutaneous thrombectomy of FEs in transit. It underscores the need for advanced therapies in the management of PE, right heart thrombi, and FE.

For patients deemed high risk of deterioration, the 2011 American Heart Association^11^ and the 2019 European Society of Cardiology guidelines^12^ recommend surgical pulmonary embolectomy or percutaneous catheter-directed treatments as an alternative in patients with contraindications to receive systemic fibrinolysis, or patients who remain unstable after thrombolysis; however, open surgical thrombectomy carries significant risks, and many patients are not suitable candidates for it.

Furthermore, fat would not respond to fibrinolysis, leaving only the open surgical option for FE.

FE adds another potential risk: the risk of FES, which carries an estimated mortality between 7% and 36%.^2^ The standard of care is supportive intensive care, with no clear benefit from anticoagulation and corticosteroids,^5^ and mechanical support devices and extracorporeal membrane oxygenation for severe cases with refractory systemic arterial hypotension and shock.^13^

FE in transit represents a challenging scenario with potential for deterioration from multiple points of view. In this case, the concurrent presence of a PFO added to the challenge.

This patient’s hemodynamic compromise was probably multifactorial; however, after adequate resuscitation, it was thought that the CIT, the presence of a profound right-to-left shunting, and already highly elevated right ventricular afterload was contributing to the instability. At the time of the decision-making process, it was unclear why the patient was so unwell, but what was clear was that she would not tolerate further insult.

We found a case report of open surgical extraction of FE in transit in an asymptomatic young trauma patient^14^; the procedure was performed prophylactically. Our patient was very symptomatic, but similarly we performed the procedure with a prophylactic intent rather than a curative one because we were not sure the volume of emboli was enough to justify the clinical presentation.

The PERT team considered all options, including expectant management potentially leading to palliation, open surgical embolectomy, and finally endovascular large-bore thrombectomy.

Our patient was not a candidate for open surgery due to her age and overall frailty. However, she was previously independent, so minimally invasive intervention was considered reasonable.

The Inari FlowTriever system has United States Food and Drug Administration approval for retrieval of CIT in the right atrium, and indeed, successful case reports can be found in the literature.^15^^,^^16^ The device was chosen for its size and ability to return blood by using the blood saver device. At the time of this case report, we did not have access to the new generation Penumbra Lightning Flash (Penumbra Inc) device.

The procedure was quick, with minimal blood loss and no complications, allowing for discharge from the ICU within 48 hours. To our knowledge, this case report is the first to demonstrate that FE in transit can be safely and effectively removed using the Inari FlowTriever system. The authors believe large-bore thrombectomy should be considered in patients with FEs in transit because expectant management can result in catastrophic consequences.

## Conclusion

This case demonstrates that percutaneous FE aspiration thrombectomy with the Inari FlowTriever system can be performed safely, is effective, and should be considered even when in transit.

## Funding

None.

## Disclosures

L.V. and A.M. have received consultancy fees from Inari in the past but not related to this manuscript.
